# Role of augmented transferrin during the retraining for undeveloped left ventricle

**DOI:** 10.1111/jcmm.12627

**Published:** 2015-06-23

**Authors:** Wei Wei, Yihe Wu, Yongquan Ying, Shoujun Li, Shengshou Hu, Hao Zhang

**Affiliations:** a State Key Laboratory of Cardiovascular Disease, National Center for Cardiovascular Disease, Fuwai Hospital, Chinese Academy of Medical Sciences, Peking Union Medical CollegeBeijing, China; b Center for Pediatric Cardiac Surgery and Research Center for Cardiac Regenerative Medicine, Fuwai Hospital, Chinese Academy of Medical Sciences and Peking Union Medical CollegeBeijing, China; c Department of Thoracic Surgery, 1st Affiliated Hospital, Zhejiang UniversityHangzhou, China

**Keywords:** transferrin, proteomics, congenital heart disease, cardiomyocyte, cardiac surgery

## Abstract

Transposition of great arteries (TGA) is a common congenital heart disease. Left ventricle (LV) is rapidly regressing and pulmonary artery banding (PAB) is utilized to retrain the undeveloped LV. Hence, it offered a unique human disease model to investigate the process of LV hypertrophy under pressure overload. Eight late referred children with TGA were enrolled. The plasma was collected at the 30 min. before and 48 hrs after PAB, and 25 proteins were identified as having significant change in proteomic analysis. Transferrin (TF) and ceruloplasmin were then confirmed. After 48 hrs incubation with TF, the size of human induced pluripotent stem cell-derived cardiomyocytes increased by two times as large as control. Meanwhile, protein synthesis and the expression of natriuretic peptide precursor A and B were significantly enhanced. TF treatment also activated both extracellular signal-regulated kinase 1/2 and activated protein kinase singling pathways. Our data provided a link to molecular components and pathways that might be involved in LV retraining. TF severed as the carrier to delivery irons, and could directly stimulate cardiomyocytes hypertrophy. TF administration may hold therapeutic potential for the biological LV retraining.

## Introduction

Transposition of the great arteries with intact ventricular septum (TGA-IVS) is a common type of cyanotic congenital heart defects, in which the aorta arises from the right ventricle and the pulmonary trunk arises from the left ventricle (LV). In patients with TGA-IVS, the pulmonary arterial pressure descends gradually and thereby the LV rapidly regressing after neonatal period. Arterial switch operation (ASO) is a standard procedure for surgical anatomical correction for TGA-IVS. Arterial switch operation is recommended to be performed in the first week or two of life. However, owing to various reasons, late referral of patients with TGA-IVS is common in China 1. Pulmonary artery banding (PAB) was proposed by Dr. Yacoub in 1976 as retraining procedure to increase LV pressure load and thereby stimulate LV hypertrophy 2. Pulmonary artery banding imposes a large pressure load on LV, and lead to series of LV changes, including hypertrophy and/or enlargement of the LV. LV retraining for TGA-IVS offered a unique human disease model to investigate the LV undergoing the process from atrophy to hypertrophy in response to a sudden dramatically increased pressure load.

Using differential gel electrophoresis (DIGE) proteomic analysis, we sought to determine the plasma protein changes that occurred in children with TGA-IVS who underwent LV retraining. And then, the underlying mechanism was investigated by a human induced pluripotent stem cell (iPSC) -derived cardiomyocytes model.

## Materials and methods

### Patients grouping

The Committee of Clinical Research in Fuwai Hospital approved this study, and informed consents were obtained from all patients’ parents.

Two millilitres plasma from the child with TGA-IVS (*n* = 8, age: 25.9 ± 19.2 months, weight: 10.3 ± 4.3 kg) was collected 30 min. before (Control group) and 48 hrs after (Test group) PAB for DIGE proteomics. An immediate increase in mean LV pressure was achieved with PAB (59.9 ± 5.1 *versus* 27.6 ± 8.8 mmHg, post- *versus* pre-banding pressures; *P* < 0.001).

For verification and validation of DIGE results, the plasma of 16 children with TGA-IVS undergoing PAB and 12 children undergoing open-chest surgery without cardiopulmonary bypass (C group and D group) were collected for ELISA. Additionally, the plasma of 12 children without congenital heart disease (E group) was used as control group.

### Proteomics analysis

Each gel consisted of three samples run concurrently: one 30 min. before PAB sample (Control group), one 48 hrs after PAB sample (Test group), and an internal standards. Plasma from Control group and Test group was labelled with either Cy3 (green) or Cy5 (red). The three samples (Cy2, Cy3, Cy5) to be run on a single gel were then combined and applied to immobilized pH gradient strips. Gels were scanned and imported into DeCyder 6.5 differential analysis software. All gels were standardized with the internal standard (Cy2) images. A visible protein stain, Coomassie Brilliant Blue G250 stain was applied to allow visual inspection of the protein spots for mass spectrometic analysis.

Upon sonication, peptides were extracted and dried completely by centrifugal lyophilization. Peptide mixtures were dissolved, and 1 mL of peptide solution was mixed with 1 mL of matrix before spotting on the Matrix-assisted laser desorption/ionization sample plate. The peptide mass fingerprinting and sequence tag data were evaluated with GPS Explorer™ software (Applied Biosystems, Foster City, CA, USA). Mass spectrometry (MS)/MS spectra were submitted to the Biotechnology Information database IPI HUMAN 3.83 to generate ion scores *via* the Mascot search engine (Matrix Science Inc, Boston, MA, USA) 3.

### ELISA and serum iron analysis

The expression levels of ceruloplasmin (CP), alpha-1-antitrypsin (SERPINA1), and transferrin (TF) were measured, respectively, with the ELISA kits. The optical density was measured at a wavelength of 450 nm by a Model 680 Microplate Reader (Thermo Fisher, Waltham, MA, USA). Serum iron, serum TF and TF saturation were measured from these blood samples by Hitachi automatic biochemistry analyzer 7100 (Hitachi, Yokohama, Japan).

### Generation, culture and differentiation of human induced pluripotent stem cells

The human fibroblasts were received as a courtesy from Dr. ZB Jin, Wenzhou Medical University. The cells were collected from the health young women who received cosmetic surgery. The fibroblasts were reprogrammed to generate iPSCs by transduction with four human reprogramming factors including Sox2, Klf4, Oct4 and c-Myc within lentiviruses as previously described 4. Induced pluripotent stem cells were differentiated into beating cardiomyocytes using a small molecule–based monolayer method 5. After differentiation, human iPSC-derived cardiomyocytes were cultured in RPMI medium plus B-27 Supplement (Life Technologies, Carlsbad, CA, USA). Spontaneously contracting cells from post-differentiation days 50–60 were used in the following experiment.

### Measurement of cardiomyocyte size after target protein treatment

Spontaneously contracting cardiomyocytes were treated with 0.1 μM endothelin (ET)-1, 5 μM albumin (Sigma-Aldrich, St Louis, MO, USA) and different doses (0.5, 2, 10 μM) of holo-TF or apo-TF (R&D Systems, Minneapolis, MN, USA) for 48 hrs. The group without additional protein treatment served as Blank group. Albumin, a high abundant protein in serum, served as negative group, and ET-1 as positive group. After 48 hrs’ incubation, cardiomyocytes were immunostained with the cardiac troponin T antibody. The nuclei were stained with 4’,6-diamidino-2-phenylindole (DAPI, Sigma-Aldrich). Total cell surface areas were calculated using NIH Image J 1.32j software (http://rsb.info.nih.gov/ij/), and expressed as the average of 350–400 randomly selected cardiomyocytes per condition.

### Real-time polymerase chain reaction

Total RNA was extracted and double-stranded complementary DNA was synthesized from 1 μg of RNA sample. Primers for natriuretic peptide precursor A (Nppa), natriuretic peptide precursor B (Nppb) and calsequestrin 2 (Casq2) were all designed by Autoprime software (http://autoprime.de). Real-time PCR was performed in a 50-μl volume reaction, The relative amount of target mRNA normalized to Casq2 was analysed by using the 2^−ΔCt^ method 6.

### Total protein & western blot

Total protein concentration of the samples was determined by use of the BCA Protein Assay Kit (Invitrogen). Thirty milligrams cellular proteins were fractionated by SDS-PAGE and transferred to Hybond membranes. The blotted membranes were incubated with a polyclonal antibody recognizing extracellular signal-regulated kinase (ERK)1/2, Akt, P38, p-ERK1/2, p-Akt, p-P38 (Cell Signaling Technology, Boston, MA, USA) and TF receptor (Abcam, Cambridge, MA, USA). Horseradish peroxidase-conjugated anti-rabbit IgG antibody was used as secondary antibody, and signals were detected using the ECL (Enhanced Chemiluminescent) detection kit.

### Statistical analysis

Data are presented as mean ± SD. If >2 groups were compared, One-way anova were used. If the test of homogeneity of variances indicated that there was heterogeneity of variance, the Welch test was used for robust tests of equality of means. The least significant difference or Dunnett’s T3 test was used followed by post hoc analysis with multiple comparison test to control the increase in the type I error. If =2 groups were compared, independent-samples *t*-test or paired-samples *t*-test were applied. *P*-values <0.05 (two-tailed) were considered statistically significant.

## Results

### Detection of differentially expressed plasma proteins

**Figure 1:**
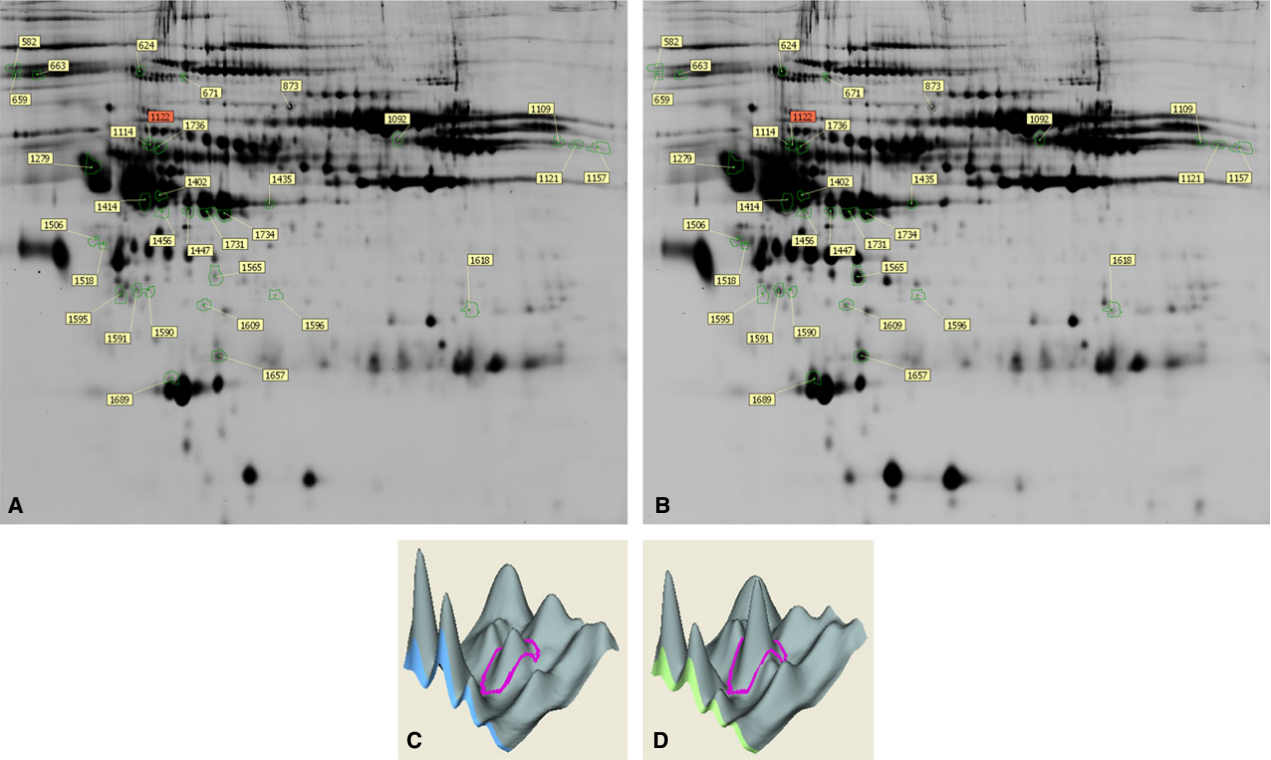
Example of 2-dimensional differential gel electrophoresis of plasma of children with TGA-IVS. The Cy3 (A, Control) and Cy5 (B, Test) images are shown including identification numbers for the protein spots of interest. A typical 3-dimensional representation of a protein gel spot, as analysed and displayed by DeCyder software, is shown below the gels, taking parvalbumin alpha (spot 1122) as an example. The Cy3 (C, Control) protein image is on the left and the Cy5 (D, Test) image on the right.

Comparison of plasma protein expression by univariate analyses found that 32 protein spots had statistically significant differential expression (*P* < 0.05). 27 spots were significantly upregulated >1.5-fold and 5 spots were significantly downregulated >1.5-fold after PAB (*P* < 0.05). A representative gel image is shown in Figure 1.

### Identification of proteins of interest by MS

From the colloidal Coomassie-stained gels, 32 differentially expressed protein spots were picked and subjected to mass spectrometry. Table 1 listed the identified protein spots as outlined in Figure 1. And 25 proteins were successfully identified by MS. All the iron ion homeostasis and heart development proteins, including CP, SERPINA1 and TF (2 spots) showed increased differential expression in the Test group, ranging from 1.54- to 1.96-fold.

**Table 1:** MS identification of differentially expressed plasma proteins spots detected by DIGE

Gel spot number*	Protein name	Accession number†	Fold change‡
Function related to iron ion homeostasis			
671	Ceruloplasmin	IPI00017601	1.56
1402	Alpha-1-antitrypsin	IPI00553177	1.54
1731	Transferrin	IPI00022463	1.59
1734	Transferrin	IPI00022463	1.96
Function related to heart development			
1122	Parvalbumin alpha	IPI00219703	1.72
Function related to protein biosynthesis and protein folding			
873	Isoform 1 of Gelsolin	IPI00026314	−1.78
1114	Mitochondrial ribosomal protein L17	IPI00982310	2.09
1657	Serum amyloid P-component	IPI00022391	2.59
Function related to substance metabolism			
1414	Glycogen phosphorylase, muscle form isoform 2	IPI00657751	2.08
1591	Apolipoprotein J	IPI00980864	1.57
1609	Apolipoprotein E	IPI00021842	2.23
1689	Apolipoprotein A-I	IPI00021841	2.19
Function related to signal transduction			
1157	Isoform 2 of Fibrinogen alpha chain	IPI00029717	2.71
1435	Fibrinogen beta chain	IPI00298497	1.97
1447	Isoform Gamma-A of Fibrinogen gamma chain	IPI00219713	1.71
1565	Haptoglobin	IPI00641737	2.96
Function related to acute-phase response and complement activation			
624	Inter-alpha (globulin) inhibitor H4	IPI00798006	2.04
663	Alpha-2-macroglobulin	IPI00478003	−1.76
1279	Alpha-1-antichymotrypsin	IPI00847635	4.23
1590	Complement C3 (Fragment)	IPI00783987	1.51
1618	Complement component 4A	IPI01012744	1.94
1736	Complement component C9	IPI00022395	1.52
Function related to others			
1092	tRNA isopentenylpyrophosphate transferase isoform 7	IPI01010521	4.82
1506	Leucine-rich alpha-2-glycoprotein	IPI00022417	2.33
1518	EVI5-like protein isoform 1	IPI00930364	2.65
1596	Single-stranded DNA binding protein 1	IPI00947310	2.34

* Spot number after spot matching across all experiments.

† Protein accession number is obtained from IPI HUMAN 3.83 database.

‡ Fold change of protein spots by median of standardized abundances in Test group *versus* Control group. A ratio of 2.0 is a twofold increase, −2.0 is a twofold decrease.

Protein spots depicted were statistically significant by the Student’s paired *t*-test (*P* < 0.05) comparing Test group protein spot volumes to corresponding Control group spot volumes after standardization to the internal control.

**Figure 2:**
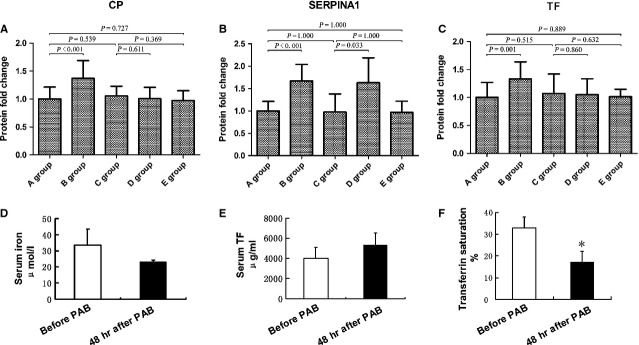
Confirmation and quantification of differentially expressed proteins in plasma. Change in the expression level of ceruloplasmin (A; CP), SERPINA1 (B) and transferrin (C; TF) were confirmed by ELISA. Serum iron (D), serum TF (E) and transferrin saturation (F) were measured before and after PAB (*P < 0.05 *versus* group before PAB). A group: the plasma of 16 children with TGA-IVS 30 min. before PAB surgery; B group: the plasma of 16 children with TGA-IVS 48 hrs after PAB surgery; C group: the plasma of 12 children 30 min. before open-chest surgery without cardiopulmonary bypass; D group: the plasma of 12 children 48 hrs after open-chest surgery without cardiopulmonary bypass; E group: the plasma of 12 children without congenital heart disease.

The three differentially expressed proteins, CP, SERPINA1 and TF were further evaluated in undepleted plasma by ELISA. ELISA analysis confirmed differential plasma levels for CP and TF (Fig. 2A–C). Serum iron level before and 48 hrs after PAB were 33.49 ± 9.96 and 23.24 ± 1.28, respectively, showing no significant differences. Forty-eight hours after PAB, serum TF level was elevated, same result as ELISA, and TF saturation was notable decreased (Fig. 2D–F).

### TF mediating the hypertrophic response in human iPSC-derived cardiomyocytes

**Figure 3:**
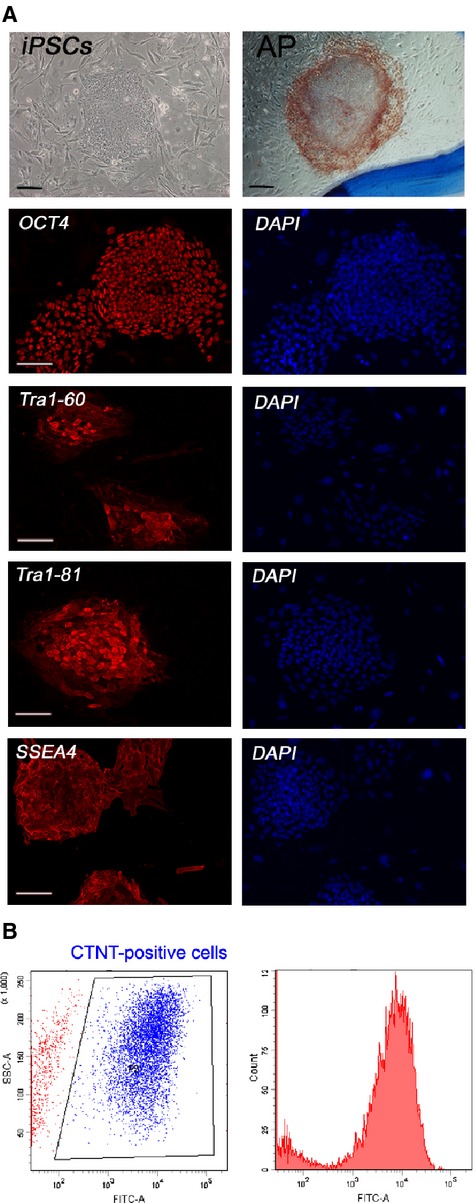
Establishment and characterization of human induced pluripotent stem cells and derived cardiomyocytes. (A) Reprogramming of human fibroblasts to iPSCs show pluripotent stem cell morphology with positive alkaline phosphatase (AP) staining and expression of pluripotency markers: OCT4, TRA1-60, TRA1-81 and SSEA4; scale bars = 100 μm. (B) Flow cytometric analysis showed cardiac specific marker troponin T (CTNT)-positive cells expressed as 89% of total cell number.

To characterize the generated iPSCs, their expression of the pluripotent markers were examined by using SSEA4, Oct4 and alkaline phosphatase (AP) staining (Fig. 3A). Immunostaining and flow cytometric analysis for iPSC-cardiomyocytes showed expression of typical cardiac markers cardiac troponin T in over 85% of the cells (Fig. 3B).

**Figure 4:**
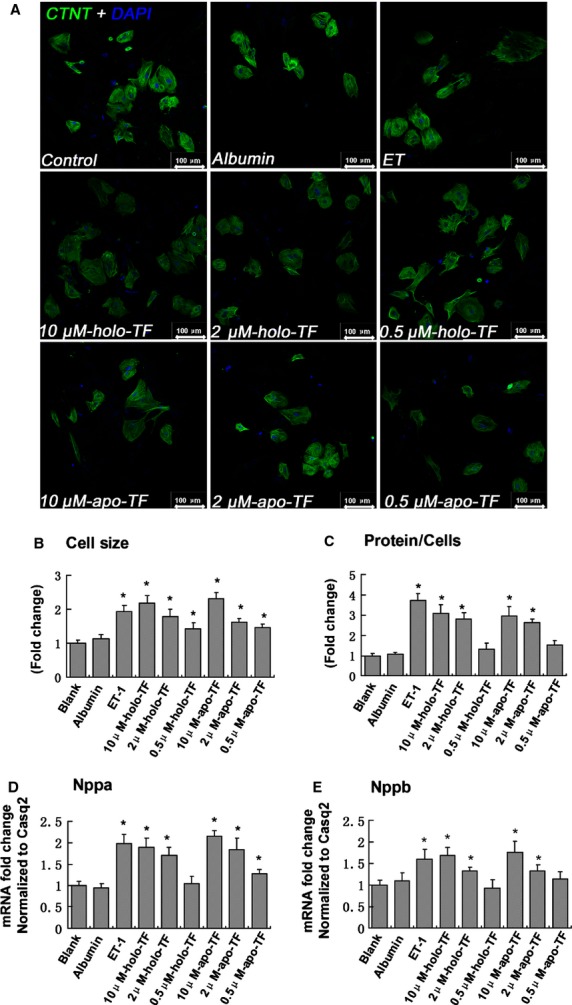
Effects of TF on human induced pluripotent stem cells derived cardiomyocyte and the expression of Nppa and Nppb mRNA levels. (A) Representative micrographs of myocytes treated with ET-1 (0.1 μM), albumin (5 μM), holo-TF or apo-TF for 48 hrs. (B) Quantification of cell size. (C) Total protein concentration of cardiomyocytes. (D and E) Quantitative results of Real-time PCR, showing Nppa and Nppb mRNA levels relative to Casq2. Data were normalized to Blank group that was arbitrarily set to 1.0. Values are means ± SD of 3 independent experiments performed in triplicates (*P < 0.05 *versus* Blank group).

Cell treatment with holo-TF dose-dependently promoted the hypertrophic response, as assessed by comparing cardiomyocyte size (10 μM-holo-TF group 2.19 ± 0.22-fold, 2 μM-holo-TF group 1.79 ± 0.21-fold, 0.5 μM-holo-TF group 1.42 ± 0.18-fold, *P* < 0.05 *versus* Blank group, Fig. 4A and B). Identical results were obtained when cardiomyocytes were treated with apo-TF (Fig. 4A and B). ET-1 was to serve as a positive control, and the 10 μM-holo-TF, 10 μM-apo-TF elicited similar effect on stimulating hypertrophy compared to ET-1. In addition, TF treatment increased protein synthesis, and the mRNA levels of Nppa and Nppb associated with cells hypertrophy (Fig. 4C–E).

**Figure 5:**
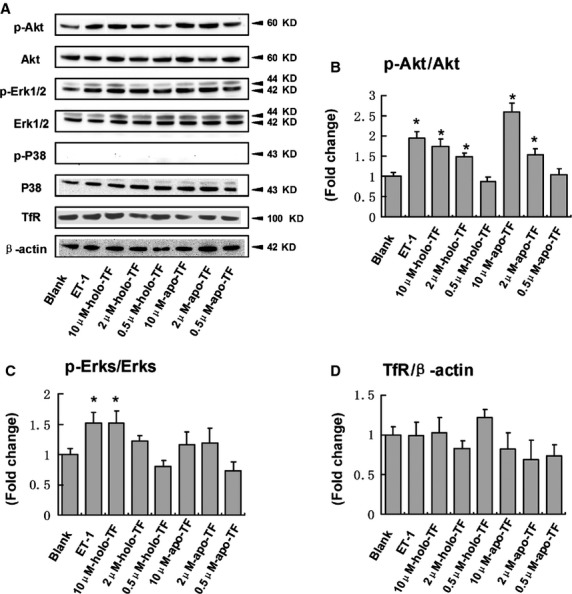
Effects of TF on p-Akt, p-ERK1/2, p-P38 and transferrin receptor(TfR) levels in human induced pluripotent stem cells derived cardiomyocytes. (A) Representative blots of cardiomyocytes treated with ET-1 (0.1 μM), holo-TF or apo-TF for 48 hrs. (B–D) Quantitative analysis. p-Akt, p-Erk1/2 and TfR levels were normalized by expressing them relative to Akt, Erk1/2 and β-actin levels, respectively. Mean values for Blank group were normalized to 1.0. Values are means ± SD (*n* = 3, *P < 0.05 *versus* Blank group).

To identify potential downstream signalling pathways activated by TF, the phosphorylation of ERK1/2 (p-ERK1/2), p-Akt and p-P38 with ET-1, holo-TF and apo-TF was measured. P-Akt and p-ERK1/2 were elevated at 48 hrs in both holo-TF and apo-TF treatment, but not p-P38 (Fig. 5A). Quantitative analysis of TF-induced Akt activation showed that 10 μM holo-TF and 10 μM apo-TF induced a significant increase in the phosphorylation level of Akt (Fig. 5B). Activation of Erk1/2 also showed dose-dependent effect, and 10 μM holo-TF induced a significant increase of p-Erk1/2, whereas apo-TF did not (Fig. 5C). Forty-eight hours exposure to ET-1 and TF did not result in significant changes in the expression of transferrin receptor (TfR) (Fig. 5D).

## Discussion

Pressure-overload induced cardiac hypertrophy has been studied intensively over the last decade. However, the hypertrophy of undeveloped heart has been neglected. Pulmonary artery banding is the first stage operation for patients with TGA/IVS who were late referred for ASO. The mechanism of LV in response to the increased workload is rather complicated 7,8. The failure of the animal model to mimic human TGA 9 makes the mechanism study for PAB very difficult. However, the overloaded LV definitely released or recruited various proteins in the peripheral blood to participate in the process of hypertrophy 10,11. Therefore, we focused on the altered plasma proteins which could mediate the hypertrophy of immature cardiomyocytes. As described by a classic study, the foetal isoforms of heavy chain myosin were re-expressed within 2 days in response to pressure overload 12. And furthermore, measurement of the rate of muscle protein synthesis demonstrated that maximal synthesis was occurring by 48 hrs from the onset of the pressure load. The clinical experiences of rapid two-stage ASO also supported this concept 13. Therefore, the plasma of TGA-IVS children was collected at the 48 hrs after PAB for the protein screening.

The major findings in the present study were: (*i*) 25 differentially expressed proteins were successfully identified by MS, and differential plasma levels of TF and CP were then confirmed. (*ii*) Both holo- and apo-TF could directly stimulate the hypertrophy of human iPSC-derived cardiomyocytes.

The identified changed proteins were involved in iron ion homeostasis, heart development, protein biosynthesis and protein folding, substance metabolism, signal transduction, acute-phase response and complement activation.

Iron plays a key role in oxygen storage (as a component of myoglobin) and oxidative metabolism in skeletal and heart muscle (as a component of oxidative enzymes) 14,15. Iron homeostasis disorder would result in cardiac eccentric hypertrophy in rats 16. Transferrin is a ubiquitous protein in all vertebrates with a central role in iron transport and metabolism 17,18. Ceruloplasmin has a potent ferroxidase activity that catalysed the oxidation of Fe^2+^ to Fe^3+^, and accelerated the binding of iron by TF 19. Left ventricle retraining would increase the LV mass by two times and therefore leaded to the dramatically increased demand for iron. Consequently, more iron homeostasis-related proteins (CP and TF) would be mobilized into peripheral blood. And a decrease in serum iron and TF saturation after PAB indicated that iron metabolism plays a central role in the development of myocytes hypertrophy. Furthermore, children with open-chest surgery and non-cardiac surgery appeared no different in TF and CP change (Fig. 2). That indicated such increase of TF and CP was not the result of inflammatory or anaemia but a phenomenon unique to PAB.

However, TF is a high abundant protein in the plasma 20. In present study, we applied human iPSC-derived cardiomyocytes model to evaluate the effect of TF. Human iPSC-derived cardiomyocytes were cultured under serum-free conditions using a B27 Supplement, which containing very low dosage of TF 21. The *in vitro* study demonstrated that treatment of human cardiomyocytes with holo- and apo-TF dose-dependently promoted the hypertrophic response, including increased cardiomyocyte size, protein synthesis and mRNA levels of hypertrophic markers. The holo- and apo-TF elicited similar stimulating effects, indicating that such a function of TF was unrelated to its iron-carrying capacity. However, the process of hypertrophy after LV retraining was very complicated and many factors would be involved. We only focused on TF based on the finding form the proteomics study, and the detailed underlying mechanism still need further investigation.

Transferrin served as a carrier to transport the irons and some proteins through TfR. Transferrin receptor also had a role in the regulation of cell proliferation, differentiation and malignant transformation 22,23. Previous report has linked iron disorder with the rapid change of TfR expression at day 8 24. In our study, TfR expression showed no significant different change after 48 hrs treatment with TF. The binding of TF to TFR on the cell surface would induce activation of mitogen-activated protein kinase and phosphatidylinositol 3-kinase signalling pathways 17. Previous studies have shown that physiological and pathological hypertrophy has distinct functio-nal characteristics and distinct signalling pathways. Akt was the best-characterized target of phosphatidylinositol 3-kinase signalling pathway, which played a critical role for the induction of physiological cardiac growth 8. Meanwhile, activation of ERK1/2 and P38 in mitogen-activated protein kinase signalling pathway had been reported in response to pathological pressure overload-induced hypertrophy 25. In present study, both Akt and Erk1/2 signalling pathways were activated after TF treatment. It indicated that both the physiological and pathological hypertrophy could be involved during the LV retraining. However, until now, we could not identify which signalling pathway dominates the process of reversal of LV atrophy after PAB.

Although PAB is an effective approach to prepare the undeveloped LV for the late referred TGA-IVS patients, it also will make the following ASO become very complicated. The administration of TF could offer more carriers to transport irons for the LV hypertrophy. Furthermore, the augmented TF could directly stimulate the cardiomyocytes hypertrophy. Hence, TF administration has the clinical implications for the late referred children with TGA/IVS.
